# Rules for Good Health

**Published:** 1890-02

**Authors:** 


### Rules for Good Health.

Be regular in your habits.

If possible go to bed at the same hour every night.

Rise in the morning soon after you awake.

A sponge bath of cold or tepid water should be followd by friction with towel or hand.

Eat plain food.

Begin your morning meal with fruit.

Don't go to work immediately after eating.

Be moderate in the use of liquids at all seasons.

It is safer to filter and boil drinking water.

Exercise in the open air whenever the weather permits.

In malarious districts do your walking in the middle of the day.

Keep the feet comfortable and well protected.

Wear woolen clothing the year round.

See that your sleeping rooms and living rooms are well ventilated, and that sewer gas does not enter them.

Brush your teeth at least twice a day, night and morning.

Don't worry, it interferes with the healthful action of the stomach.

You must have interesting occupation in vigorous old age. Continue to keep the brain active. Rest means rust.—*Herald of Health.*

